# Supplementary material for: Associations between police harassment and distrust in and reduced access to healthcare among Black sexual minority men: A longitudinal analysis of HPTN 061
Source: PLoS One. 2023 Aug 18;18(8):e0290378. doi: 10.1371/journal.pone.0290378 (PMC10437825; doi:10.1371/journal.pone.0290378)
Supplement: S1 Appendix — (DOCX) [file pone.0290378.s001.docx]

| Distrust in Healthcare Providers | Odds ratio | Std. err. | z | P>\|z\| | [95% conf. interval] | |
| --- | --- | --- | --- | --- | --- | --- |
| **Police Harassment** | **1.462518** | **.1788758** | **3.11** | **0.002** | **1.150783** | **1.858698** |

**Appendix/Supplementary Material: Full Models for Tables 3a and 3b for Healthcare Outcomes**

**Table S1: Bivariable Analysis: Distrust in Healthcare Providers (at six month follow-up visit)**

**Table S2: Multivariable Analysis: Distrust in Healthcare Providers (at six month follow-up visit)**

| Distrust in Healthcare Providers | Odds ratio | Std. err. | z | P>\|z\| | [95% conf. interval] | |
| --- | --- | --- | --- | --- | --- | --- |
|  |  |  |  |  |  |  |
| **Police Harassment** | **1.317569** | **.1852352** | **1.96** | **0.050** | **1.00024** | **1.735572** |
|  |  |  |  |  |  |  |
| Age Group |  |  |  |  |  |  |
| 31-50 | 1.249228 | .2621641 | 1.06 | 0.289 | .8279569 | 1.884845 |
| 51+ | 1.886535 | .5252446 | 2.28 | 0.023 | 1.093139 | 3.255775 |
| Insufficient Income | 1.34766 | .2433015 | 1.65 | 0.098 | .9460361 | 1.919787 |
| Transgender | 1.805733 | .6252786 | 1.71 | 0.088 | .916019 | 3.559611 |
| Unstable Housing | 1.061024 | .2877487 | 0.22 | 0.827 | .6235625 | 1.805386 |
| Have sex with men and women | 1.200556 | .2254876 | 0.97 | 0.330 | .8308264 | 1.73482 |
| Education | .762256 | .1345952 | -1.54 | 0.124 | .5392624 | 1.077461 |
| City of Residence |  |  |  |  |  |  |
| Atlanta | 1.571713 | .5153128 | 1.38 | 0.168 | .8265943 | 2.988506 |
| New York City | 1.175434 | .381343 | 0.50 | 0.618 | .6223651 | 2.219992 |
| Boston | 1.077501 | .3844407 | 0.21 | 0.834 | .5354494 | 2.168289 |
| Los Angeles | 1.279055 | .4172638 | 0.75 | 0.451 | .674844 | 2.424238 |
| San Francisco | 1.414879 | .5223176 | 0.94 | 0.347 | .6862607 | 2.917087 |
| HIV serostatus | 1.127626 | .243312 | 0.56 | 0.578 | .7387512 | 1.721202 |
| STI Infection | 1.631943 | .4161567 | 1.92 | 0.055 | .9900172 | 2.690092 |
| Baseline Healthcare Distrust | 3.358056 | .6067592 | 6.70 | 0.000 | 2.356602 | 4.785084 |
| Incarceration History | 1.044271 | .1992591 | 0.23 | 0.820 | .7184454 | 1.517862 |

**Table S3:Bivariate Analysis: Emergency Room Visit (at six month follow-up visit)**

| Emergency Room Use | Odds ratio | Std. err. | z | P>\|z\| | [95% conf. interval] | |
| --- | --- | --- | --- | --- | --- | --- |
| **Police Harassment** | **1.300846** | **.1677317** | **2.04** | **0.041** | **1.010349** | **1.674867** |

**Table S4: Multivariable Analysis: Emergency Room Visit (at six month follow-up visit)**

| Emergency Room Use | Odds ratio | Std. err. | z | P>\|z\| | [95% conf. interval] | |
| --- | --- | --- | --- | --- | --- | --- |
|  |  |  |  |  |  |  |
| **Police Harassment** | **1.168498** | **.2131178** | **0.85** | **0.393** | **.8172986** | **1.670611** |
|  |  |  |  |  |  |  |
| Age Group |  |  |  |  |  |  |
| 31-50 | .7920066 | .2227876 | -0.83 | 0.407 | .4563415 | 1.374572 |
| 51+ | .869285 | .3078907 | -0.40 | 0.692 | .4341866 | 1.740396 |
| Insufficient Income | .952327 | .2233794 | -0.21 | 0.835 | .6013494 | 1.508153 |
| Transgender | 1.759809 | .801334 | 1.24 | 0.215 | .720889 | 4.295983 |
| Unstable Housing | .9024756 | .3029659 | -0.31 | 0.760 | .4673933 | 1.742563 |
| Have sex with men and women | 1.377827 | .3354423 | 1.32 | 0.188 | .8549941 | 2.220373 |
| Education | .8154152 | .1879281 | -0.89 | 0.376 | .5190427 | 1.281016 |
| City of Residence |  |  |  |  |  |  |
| Atlanta | 1.354334 | .5910134 | 0.70 | 0.487 | .5758011 | 3.185509 |
| New York City | .9011204 | .3669744 | -0.26 | 0.798 | .4056369 | 2.001834 |
| Boston | 1.384783 | .5722907 | 0.79 | 0.431 | .6160338 | 3.112853 |
| Los Angeles | .8573419 | .3758587 | -0.35 | 0.726 | .3630674 | 2.024514 |
| San Francisco | 1.177789 | .5477506 | 0.35 | 0.725 | .4733714 | 2.930442 |
| HIV serostatus | .5269563 | .1679783 | -2.01 | 0.044 | .2821216 | .9842669 |
| STI Infection | .7937122 | .3188741 | -0.58 | 0.565 | .3611542 | 1.74435 |
| Emergency Room Use (baseline) | 2.488495 | .5627341 | 4.03 | 0.000 | 1.597537 | 3.876347 |
| Incarceration History | 1.389839 | .3607351 | 1.27 | 0.205 | .8356678 | 2.311508 |

**Table S5: Bivariate Analysis: Missed Healthcare Visits(at six month follow-up visit)**

| Missed Healthcare Visits | Odds ratio | Std. err. | z | P>\|z\| | [95% conf. interval] | |
| --- | --- | --- | --- | --- | --- | --- |
| **Police Harassment** | **1.500089** | **.3051377** | **1.99** | **0.046** | **1.006865** | **2.234922** |
| _cons | .0578022 | .0200323 | -8.23 | 0.000 | .0293053 | .1140098 |

**Table S6: Multivariable Analysis: Missed Healthcare Visits (at six month follow-up visit)**

| Missed Healthcare Visits | Odds ratio | Std. err. | z | P>\|z\| | [95% conf. interval] | |
| --- | --- | --- | --- | --- | --- | --- |
|  |  |  |  |  |  |  |
| **Police Harassment** | **1.929326** | **.5651711** | **2.24** | **0.025** | **1.086569** | **3.425738** |
|  |  |  |  |  |  |  |
| Age Group |  |  |  |  |  |  |
| 31-50 | .634 | .2405294 | -1.20 | 0.230 | .3014096 | 1.333587 |
| 51+ | .467703 | .2577988 | -1.38 | 0.168 | .1587765 | 1.377698 |
| Insufficient Income | 1.863512 | .6403508 | 1.81 | 0.070 | .9502492 | 3.654492 |
| Transgender | 1.287292 | .7796152 | 0.42 | 0.677 | .3927973 | 4.218765 |
| Unstable Housing | 1.105392 | .544712 | 0.20 | 0.839 | .4207881 | 2.903815 |
| Have sex with men and women | .6683172 | .2297352 | -1.17 | 0.241 | .3407066 | 1.310946 |
| Education | 1.186742 | .3856266 | 0.53 | 0.598 | .6277141 | 2.243626 |
| City of Residence |  |  |  |  |  |  |
| Atlanta | 1.33475 | .8968873 | 0.43 | 0.667 | .3576297 | 4.981569 |
| New York City | 1.220595 | .6675095 | 0.36 | 0.715 | .417901 | 3.565086 |
| Boston | .807327 | .4735716 | -0.36 | 0.715 | .2557062 | 2.548928 |
| Los Angeles | 1.627577 | .9600705 | 0.83 | 0.409 | .5121974 | 5.171849 |
| San Francisco | .9283169 | .6064975 | -0.11 | 0.909 | .2579767 | 3.340504 |
| HIV serostatus | .49981 | .2365025 | -1.47 | 0.143 | .1977103 | 1.263516 |
| STI Infection | .6928212 | .4204223 | -0.60 | 0.545 | .2109063 | 2.275898 |
| Missed Healthcare Visits (baseline) | 1.335574 | .5282416 | 0.73 | 0.464 | .6151817 | 2.899561 |
| Incarceration History | 1.622359 | .6099994 | 1.29 | 0.198 | .7764244 | 3.389962 |

**Table S7: Bivariate Analysis: Seeing a Healthcare Provider (at six month follow-up visit)**

| Seeing a Healthcare Provider | Odds ratio | Std. err. | z | P>\|z\| | [95% conf. interval] | |
| --- | --- | --- | --- | --- | --- | --- |
| **Police Harassment** | **1.140414** | **.1056767** | **1.42** | **0.156** | **.9510113** | **1.367538** |

**Table S8: Multivariable Analysis: Seeing a Healthcare Provider (at six month follow-up visit)**

| Seeing a Healthcare Provider | Odds ratio | Std. err. | z | P>\|z\| | [95% conf. interval] | |
| --- | --- | --- | --- | --- | --- | --- |
|  |  |  |  |  |  |  |
| **Police Harassment** | **1.098137** | **.1209493** | **0.85** | **0.395** | **.8849215** | **1.362725** |
|  |  |  |  |  |  |  |
| Age Group |  |  |  |  |  |  |
| 31-50 | 1.360148 | .2297959 | 1.82 | 0.069 | .9767345 | 1.89407 |
| 51+ | 1.618506 | .4137856 | 1.88 | 0.060 | .9806117 | 2.671355 |
| Insufficient Income | .9016199 | .1382459 | -0.68 | 0.499 | .6675875 | 1.217696 |
| Transgender | 2.423043 | .9927425 | 2.16 | 0.031 | 1.085466 | 5.408864 |
| Unstable Housing | 1.267148 | .3279391 | 0.91 | 0.360 | .7630196 | 2.104356 |
| Have sex with men and women | .9781067 | .1616057 | -0.13 | 0.893 | .7075355 | 1.352148 |
| Education | 1.161393 | .1759283 | 0.99 | 0.323 | .8630558 | 1.562858 |
|  |  |  |  |  |  |  |
| City of Residence |  |  |  |  |  |  |
| Atlanta | .6043528 | .1538169 | -1.98 | 0.048 | .3669839 | .9952544 |
| New York City | 1.605552 | .4027334 | 1.89 | 0.059 | .9819983 | 2.625053 |
| Boston | 2.055948 | .5903118 | 2.51 | 0.012 | 1.171148 | 3.609213 |
| Los Angeles | 1.398084 | .3569793 | 1.31 | 0.189 | .8476018 | 2.30608 |
| San Francisco | 1.436926 | .424832 | 1.23 | 0.220 | .8049593 | 2.565045 |
| HIV serostatus | 4.8381 | 1.113442 | 6.85 | 0.000 | 3.081619 | 7.59575 |
| STI Infection | .9305411 | .2034548 | -0.33 | 0.742 | .6062164 | 1.428379 |
| Seeing a Healthcare Provider (baseline) | 2.957563 | .4379382 | 7.32 | 0.000 | 2.212551 | 3.953437 |
| Incarceration History | .8993281 | .1476864 | -0.65 | 0.518 | .651831 | 1.240799 |

**Table S9: Bivariate Analysis: Distrust in Healthcare Providers (at twelve month follow-up visit)**

| Distrust in Healthcare Providers | Odds ratio | Std. err. | z | P>\|z\| | [95% conf. interval] | |
| --- | --- | --- | --- | --- | --- | --- |
| **Police Harassment** | **1.363957** | **.1834625** | **2.31** | **0.021** | **1.047871** | **1.77539** |
| _cons | .1271461 | .0285275 | -9.19 | 0.000 | .081907 | .1973718 |

**Table S10: Multivariable Analysis: Distrust in Healthcare Providers (at twelve month follow-up visit)**

| Distrust in Healthcare Providers | Odds ratio | Std. err. | z | P>\|z\| | [95% conf. interval] | |
| --- | --- | --- | --- | --- | --- | --- |
|  |  |  |  |  |  |  |
| **Police Harassment** | **1.148789** | **.1748221** | **0.91** | **0.362** | **.8525207** | **1.548017** |
|  |  |  |  |  |  |  |
| Age Group |  |  |  |  |  |  |
| 31-50 | 1.067917 | .245734 | 0.29 | 0.775 | .680254 | 1.676502 |
| 51+ | .8702531 | .289993 | -0.42 | 0.677 | .4528988 | 1.672207 |
| Insufficient Income | .9381929 | .187121 | -0.32 | 0.749 | .6346312 | 1.386956 |
| Transgender | 1.602583 | .606781 | 1.25 | 0.213 | .7630148 | 3.365955 |
| Unstable Housing | 1.640082 | .462701 | 1.75 | 0.079 | .9434608 | 2.851064 |
| Have sex with men and women | 1.078276 | .2291373 | 0.35 | 0.723 | .7109628 | 1.635359 |
| Education | .5604244 | .1114936 | -2.91 | 0.004 | .3794677 | .8276741 |
| City of Residence |  |  |  |  |  |  |
| Atlanta | 1.984726 | .7455325 | 1.82 | 0.068 | .9505158 | 4.144209 |
| New York City | 1.899108 | .6885245 | 1.77 | 0.077 | .9331365 | 3.865041 |
| Boston | 1.510886 | .6133418 | 1.02 | 0.309 | .681848 | 3.347924 |
| Los Angeles | .7453142 | .3006598 | -0.73 | 0.466 | .3380374 | 1.64329 |
| San Francisco | 1.175124 | .5234184 | 0.36 | 0.717 | .490846 | 2.813338 |
| HIV serostatus | 1.135185 | .2719757 | 0.53 | 0.597 | .7097895 | 1.81553 |
| STI Infection | .9296391 | .2846528 | -0.24 | 0.812 | .5101277 | 1.694142 |
| Baseline Healthcare Distrust | 2.406673 | .4977196 | 4.25 | 0.000 | 1.604655 | 3.609545 |
| Incarceration History | 1.364255 | .2976207 | 1.42 | 0.155 | .8896124 | 2.092139 |

**Table S11: Bivariate Analysis: Emergency Room Visit (at twelve month follow-up visit)**

| Emergency Room Visit | Odds ratio | Std. err. | z | P>\|z\| | [95% conf. interval] | |
| --- | --- | --- | --- | --- | --- | --- |
| **Police Harassment** | **1.695429** | **.2415087** | **3.71** | **0.000** | **1.282415** | **2.241459** |
| _cons | .1737033 | .0414985 | -7.33 | 0.000 | .1087557 | .2774368 |

**Table S12: Multivariable Analysis: Emergency Room Visit (at twelve month follow-up visit)**

| Emergency Room Visit | Odds ratio | Std. err. | z | P>\|z\| | [95% conf. interval] | |
| --- | --- | --- | --- | --- | --- | --- |
|  |  |  |  |  |  |  |
| **Police Harassment** | **1.355298** | **.2529063** | **1.63** | **0.103** | **.9401468** | **1.953772** |
|  |  |  |  |  |  |  |
| Age Group |  |  |  |  |  |  |
| 31-50 | .8150513 | .2215747 | -0.75 | 0.452 | .4783906 | 1.388632 |
| 51+ | .5101914 | .1867344 | -1.84 | 0.066 | .2489924 | 1.045395 |
| Insufficient Income | 1.01609 | .2387287 | 0.07 | 0.946 | .6411267 | 1.610351 |
| Transgender | .8574638 | .4526965 | -0.29 | 0.771 | .304667 | 2.413271 |
| Unstable Housing | .7920967 | .2883271 | -0.64 | 0.522 | .3880938 | 1.616664 |
| Have sex with men and women | 1.21314 | .299773 | 0.78 | 0.434 | .7474371 | 1.969006 |
| Education | .9756471 | .2295553 | -0.10 | 0.917 | .6152018 | 1.547276 |
| City of Residence |  |  |  |  |  |  |
| Atlanta | 1.654489 | .7117361 | 1.17 | 0.242 | .7120159 | 3.844482 |
| New York City | 1.446122 | .5812525 | 0.92 | 0.359 | .6577703 | 3.179332 |
| Boston | 1.717765 | .7359862 | 1.26 | 0.207 | .7417571 | 3.97801 |
| Los Angeles | 2.040476 | .875012 | 1.66 | 0.096 | .8804665 | 4.72879 |
| San Francisco | 1.462717 | .6936576 | 0.80 | 0.423 | .5774297 | 3.705286 |
| HIV serostatus | .5782954 | .1763905 | -1.80 | 0.073 | .3180665 | 1.051433 |
| STI Infection | 1.528371 | .5574765 | 1.16 | 0.245 | .7477413 | 3.123966 |
| Emergency Room Visit (baseline) | 2.695249 | .6149503 | 4.35 | 0.000 | 1.723408 | 4.215119 |
| Incarceration History | 1.333126 | .3406647 | 1.13 | 0.261 | .8078985 | 2.199813 |

**Table S13: Bivariate Analysis: Missed Healthcare Visits (at twelve month follow-up visit)**

| Missed Healthcare Visits | Odds ratio | Std. err. | z | P>\|z\| | [95% conf. interval] | |
| --- | --- | --- | --- | --- | --- | --- |
| **Police Harassment** | **1.141311** | **.2299416** | **0.66** | **0.512** | **.7689728** | **1.693935** |

**Table S14: Multivariable Analysis: Missed Healthcare Visits (at twelve month follow-up visit)**

| Missed Healthcare Visits | Odds ratio | Std. err. | z | P>\|z\| | [95% conf. interval] | |
| --- | --- | --- | --- | --- | --- | --- |
|  |  |  |  |  |  |  |
| **Police Harassment** | **1.037305** | **.2779707** | **0.14** | **0.891** | **.6134888** | **1.753906** |
|  |  |  |  |  |  |  |
| Age Group |  |  |  |  |  |  |
| 31-50 | 1.595513 | .6734319 | 1.11 | 0.268 | .6976316 | 3.649004 |
| 51+ | .2669856 | .2218621 | -1.59 | 0.112 | .052378 | 1.360902 |
| Insufficient Income | 2.087364 | .760386 | 2.02 | 0.043 | 1.022167 | 4.262597 |
| Transgender | .2923425 | .3160795 | -1.14 | 0.255 | .0351218 | 2.433367 |
| Unstable Housing | .6084175 | .4063121 | -0.74 | 0.457 | .1643441 | 2.25242 |
| Have sex with men and women | .88409 | .3291463 | -0.33 | 0.741 | .4261814 | 1.833996 |
| Education | .9652997 | .3358485 | -0.10 | 0.919 | .4881023 | 1.909033 |
| City of Residence |  |  |  |  |  |  |
| Atlanta | 2.066349 | 1.312008 | 1.14 | 0.253 | .5953075 | 7.172423 |
| New York City | 1.013154 | .5431174 | 0.02 | 0.981 | .3543036 | 2.897181 |
| Boston | .6938259 | .4154065 | -0.61 | 0.542 | .2145953 | 2.243266 |
| Los Angeles | .7722563 | .5425834 | -0.37 | 0.713 | .194855 | 3.060634 |
| San Francisco | 1.290241 | .8301211 | 0.40 | 0.692 | .3656125 | 4.553242 |
| HIV serostatus | .7493469 | .3403641 | -0.64 | 0.525 | .3076486 | 1.825202 |
| STI Infection | .3973436 | .259759 | -1.41 | 0.158 | .1103323 | 1.430967 |
| Missed Healthcare Visits (baseline) | 3.610617 | 1.395798 | 3.32 | 0.001 | 1.692476 | 7.702654 |
| Incarceration History | .7458206 | .2789789 | -0.78 | 0.433 | .358292 | 1.5525 |

**Table S15: Bivariate Analysis: Seeing a Healthcare Provider (at twelve month follow-up visit)**

| Seeing a Healthcare Provider | Odds ratio | Std. err. | z | P>\|z\| | [95% conf. interval] | |
| --- | --- | --- | --- | --- | --- | --- |
| **Police Harassment** | **1.088315** | **.1075056** | **0.86** | **0.392** | **.8967503** | **1.320802** |

**Table S16: Multivariable Analysis: Seeing a Healthcare Provider (at twelve month follow-up visit)**

| Seeing a Healthcare Provider | Odds ratio | Std. err. | z | P>\|z\| | [95% conf. interval] | |
| --- | --- | --- | --- | --- | --- | --- |
|  |  |  |  |  |  |  |
| **Police Harassment** | **1.057913** | **.1234372** | **0.48** | **0.629** | **.8416501** | **1.329744** |
|  |  |  |  |  |  |  |
| Age Group |  |  |  |  |  |  |
| 31-50 | 1.347339 | .2391974 | 1.68 | 0.093 | .9513935 | 1.908066 |
| 51+ | 2.504095 | .7172574 | 3.20 | 0.001 | 1.428361 | 4.389991 |
| Insufficient Income | .9308074 | .1882213 | -0.35 | 0.723 | .6262328 | 1.383515 |
| Transgender | 1.509204 | .8071211 | 0.77 | 0.442 | .5290854 | 4.304967 |
| Unstable Housing | .9167638 | .3305554 | -0.24 | 0.810 | .4522116 | 1.858546 |
| Have sex with men and women | .7025557 | .1538499 | -1.61 | 0.107 | .4573825 | 1.07915 |
| Education | .8937573 | .1826606 | -0.55 | 0.583 | .5987648 | 1.334083 |
| City of Residence |  |  |  |  |  |  |
| Atlanta | .4864173 | .1748388 | -2.01 | 0.045 | .2404648 | .9839355 |
| New York City | 1.129091 | .3610613 | 0.38 | 0.704 | .6032968 | 2.113132 |
| Boston | 1.13603 | .4119571 | 0.35 | 0.725 | .5581095 | 2.312384 |
| Los Angeles | .6155487 | .2054142 | -1.45 | 0.146 | .3200433 | 1.183903 |
| San Francisco | .9500012 | .3718755 | -0.13 | 0.896 | .4410862 | 2.04609 |
|  |  |  |  |  |  |  |
| HIV serostatus | 4.909204 | 1.849776 | 4.22 | 0.000 | 2.345742 | 10.27405 |
| STI Infection | .6744046 | .1958081 | -1.36 | 0.175 | .3817515 | 1.191407 |
| Seeing a Healthcare Provider (baseline) | 1.03017 | .302422 | 0.10 | 0.919 | .5794635 | 1.831437 |
| Incarceration History | .8449747 | .2496247 | -0.57 | 0.569 | .4735649 | 1.507676 |
