# Supplementary material for: Associations between police harassment and distrust in and reduced access to healthcare among Black sexual minority men: A longitudinal analysis of HPTN 061
Source: PLoS One. 2023 Aug 18;18(8):e0290378. doi: 10.1371/journal.pone.0290378 (PMC10437825; doi:10.1371/journal.pone.0290378)
Supplement: Attachment — Submitted filename: Reviewer_response_PLOS_09182022.docx [file pone.0290378.s002.docx]

Reviewer #1: Thank you for the opportunity to review the manuscript entitled “Associations between Police Harassment and Distrust in and Reduced Access to Healthcare Among Black Sexual Minority Men: A Longitudinal Analysis of HPTN 061.” This manuscript examines identity-based police harassment (IBPH) as a predictor of 1) distrust in, and 2) reduced engagement in healthcare for Black men who have sex with men (BMSM).

This is a very important area of study, and the authors are to be commended for these data.
However, a few concerns lead me not to recommend publication at this time. Most importantly, I am not confident that the results support the general conclusions. Section-specific comments are appended below.

Introduction
The authors do a nice job of contextualizing the study. BMSM face disparities in a variety of health domains. Low engagement with healthcare may be an important explanation for these disparities, with established barriers, such as medical mistrust. These could be addressed if we know more about their sources. As such, the research question: what broader socio-structural factors contributes to those barriers?

However, it would be helpful for the reader to have the relationships between specific variables spelled out and justified.

**Author response: We thank the reviewer for the careful review of our important paper and highlighting key strengths of the analysis. We have addressed the reviewer’s comments below as well as in the revised manuscript and, importantly, feel that the paper has been strengthened as a result. We have detailed the relationships between the different variables further in the introduction and discussions sections of the manuscript, and we have additionally highlighted some of the limitations of some of the outcome variables as well (for instance, the use of ED department outcome variable). We have also drawn on previous literature that has evaluated similar relationships between IBPH and healthcare outcomes to further justify the analysis in the manuscript (see Alang, S., McAlpine, D. D., & Hardeman, R. (2020). Police brutality and mistrust in medical institutions. *Journal of racial and ethnic health disparities, 7*(4), 760-768)**

1) Could the authors elaborate on the reasoning why IBPH is likely highly prevalent for BMSM?

**Author response: As suggested by the reviewer, we have added a paragraph in the introduction that has introduced several factors associated with IBPH among racial and sexual minority populations, highlighting the disproportionate rates of police encounters among these populations, and some of the negative outcomes associated with these encounters including increased stress and mental health symptoms. We have included several important references as well that have highlighted these disparities in previous research on these particular populations.**

**To summarize we note BSMM experience elevated rates of IBPH for a number of reasons. First, racial minority populations face disproportionate levels of discrimination in the criminal justice system, leading to higher rates of police encounters. Second, compared to their white counterparts, minority populations who face police encounters are more likely to experience aggressive physical contact and harsh language from law enforcement, and there is evidence that sexual minorities are treated more disrespectfully when stopped by police, possibly due to transgressions in gender norms. Among sexual minority populations, nearly half who have had encounters with police have reported police misconduct, which can lead to stress and mental health problems among these populations.**

**Here is a list of relevant references that were included in the introduction highlighting this information:**

**Brunson, R. K., & Weitzer, R. (2009). Police relations with black and white youths in different urban neighborhoods. *Urban Affairs Review, 44*(6), 858-885.**

**Davis, E., Whyde, A., & Langton, L. (2018). Contacts between police and the public, 2015. *US Department of Justice Office of Justice Programs Bureau of Justice Statistics Special Report*, 1-33.**

**Fagan, J., Geller, A., Davies, G., & West, V. (2009). *Street Stops and Broken Windows Revisited: The Demography and Logic of Proactive Policing in a Safe and Changing City*. Retrieved from New York, NY:**

**Geller, A. (2017). Policing America’s children: Police contact and consequences among teens in fragile families. *IDEAS Work Pap Ser from RePEc*, 1-46.**

**Mallory, C., Hasenbush, A., & Sears, B. (2015). Discrimination and harassment by law enforcement officers in the LGBT community.**

2) Could the authors describe the theoretical link between IBPH and medical distrust? The authors introduce systems avoidance, and that the spectacle of police violence could foster distrust in systems more broadly. At a second-hand level, this explanation makes sense. But, how would this play out at the intra-individual level, such that the actual experience changes intra-individual psychological processes? There is some speculation in the discussion, but it would be helpful to anticipate in the introduction.

Finally, the idea that an emergency department (ED) visit is the less stigmatizing alternative to healthcare provider (HCP) is not spelled out. Why is this engagement with healthcare considered the alternative to PCP visit rather than opting out altogether?

**Author response: In the revised manuscript, we have provided further information on the theoretical link between IBPH and medical distrust, and highlighted findings from some recent articles that has explored links between police harassment and medical mistrust. For example, in the revised manuscript, we state that “broader experiences of discrimination outside of the healthcare setting (which may include disproportionate police harassment among racial minorities) could be directly associated with mistrust in the healthcare system.” (page 5). They also note that these negative social experiences, which may include IBPH, can shape not only health status, but healthcare encounters including seeing one’s healthcare provider. We have added some context to the introduction that introduces this research and the implication that IBPH has on medical mistrust in the healthcare setting.**

**With respect to emergency department use, we highlight in the introduction that because primary medical doctors often have charts with extensive patient history, some individuals may opt to utilize ED as a way of “systems avoidance” such that system avoidance keeps justice-involved individuals away from healthcare institutions or more informal support, and may thus cause them to rely on emergency departments for health needs that could otherwise have been treated in primary care settings. As many who are justice involved often have instances of police contact, this would play a role in their overall system avoidance and lead them to utilizing the ED as part of this avoidance of their primary doctors. We have added additional language to note that because ED are not as likely to have full historical medical records for individuals, this may lead persons to choose to visit the ED rather than their regular doctor to avoid the stigma they may have experienced at their primary doctor’s office. However, there still remains some stigma in any type of medical encounter, including ED visits, which is why we chose to explore this outcome in our analysis. Furthermore, there is evidence that if individuals visit the ED as a result of the direct police encounter and violence, may lead to further stigmatization of the individuals who experience these police related injuries, particularly if they visit the ED often due to violence that is a direct result of police encounters.**

**We drew information from the references included in the manuscript, also noted below:**

**Alang, S., McAlpine, D. D., & Hardeman, R. (2020). Police brutality and mistrust in medical institutions. *Journal of racial and ethnic health disparities, 7*(4), 760-768.**

**Hammond, W. P. (2010). Psychosocial correlates of medical mistrust among African American men. *American Journal of Community Psychology, 45*(1), 87-106.**

Methods

The authors do a nice job describing the sample, their variables, and their construction. Ordinal predictor variable of IBPH is explained well. However, there is a detail about the dichotomization of the predictor variable for mediation is confusing. Further, mediation analyses are not discussed later in the manuscript.

**Author response: We had previously proposed performing a mediation analysis for this paper but after running several models we did not note strong mediating effects and the explanation of the mediation analysis would have made the paper much longer than the journal would allow, so we therefore decided to remove the mediation analysis and only present the multivariable effects of police harassment and healthcare outcomes. We have removed all mentions of mediation analysis in the updated draft and thank the reviewer for pointing this out in the methods section.**

Relatedly, the conceptual framing of the variables places distrust, HCP non-use, and increased ED use as related outcomes of IBHP. Did the authors examined distrust and non-use as mediators of the association between IBPH and increased emergency room visit?

**Author response: We performed a separate mediation analysis that examined these factors; the results of this analysis did not show any meaningful indirect effects, with no statistically significant findings in the mediation analysis results. We chose not to report this information in the manuscript and will likely perform a more detailed mediation analysis in a subsequent paper.**

Most importantly, it is clear from the description in the methods that these outcomes are tiered. Namely, data regarding the outcomes of HCP mistrust and missing visits are only collected from participants who did see a HCP in the past 6 months. This detail should be carried throughout the manuscript, as it does impact the conclusions from the study. What about those who did not see a HCP? Couldn’t this be due to mistrust from IBPH? Could those participants be analyzed as well somehow?

**Author response: We thank the reviewer for highlighting this aspect of healthcare provider visits. The current methods note that the missed visits variable was only asked of those who had seen a healthcare provider in the last six months. The participants in this study did report several co-morbidities that may lead to needing to see a healthcare provider (such as HIV and STI infection); however, there were also a number of individuals who did not have any of these conditions, and may not have had a need to visit a healthcare provider outside of regular yearly screenings/physicals. In the discussion, we have updated the manuscript to note that those who did not visit a healthcare provider may have been healthy and did not need to see one, and it could also be an artifact of having experienced police harassment recently as well. We feel these new additions have significantly improved our manuscript.**

Results
Can the authors provide p-values for all models and regression coefficients?
In unadjusted models, there was evidence that IBPH predicted:
1) at 6M, distrust in HCP, missing more than 50% of appointments, and visiting the ED.
2) at 12M, distrust in HCP, visiting the ED.

However, in adjusted analyses, the confidence intervals for all the predictors in the logistic regressions include 1.0 (except for one predictor), which suggests non-significance of the key relationships of interest. Could the authors report all p-values? These models suggest that the hypothesized relationships are due to various covariates. Can the authors report these full models, as well?

**Author response: As suggested by the reviewer, we have updated the manuscript to include the full models in the supplementary material. As we ran separate models for each of the outcomes, it would be excessive to report all of these models in the main text, but an Appendix will be included that has all covariates listed with the coefficients, p-values, and confidence intervals**.

Aside from this point, this sample reported a very high rate of previous incarceration (60.1%). I wonder if this particular factor could underpin the outcomes of interest rather than incidences of IBPH. Did the authors run models with incarceration (60.1%)as a covariate or predictor?

**Author response: Thank you for this suggestion. In the revised manuscript, we updated the analysis to include incarceration as a covariate in the multivariable models, and the updated analysis to include these effects along with the effect of incarceration in the multivariable models (which will be presented in the Appendix/Supplementary materials).**
