# Supplementary material for: Associations between police harassment and distrust in and reduced access to healthcare among Black sexual minority men: A longitudinal analysis of HPTN 061
Source: PLoS One. 2023 Aug 18;18(8):e0290378. doi: 10.1371/journal.pone.0290378 (PMC10437825; doi:10.1371/journal.pone.0290378)
Supplement: Attachment — Submitted filename: Reviewer Comments-Round 2_Final.docx [file pone.0290378.s003.docx]

**PLOS One Police Harassment and Healthcare Outcomes Paper**

Reviewer #2: The study is novel and fills an important gap in the literature on the role of structural factors in explaining differential health outcomes among racial/ethnic and sexual and gender minority populations. Overall, I found the findings compelling and the authors should be commended for their intellectual rigor and theoretical framing of this question. However, I have one larger philosophical question regarding how discrimination is being conceptualized and a couple minor issues which will be more easily addressed by the authors.

1. I would recommend the authors consider being more explicit about what it is you are measuring here. You are examining exposures of racism and discrimination among Black sexual minority populations and operationalizing this as negative police encounters. However, the way this is currently written the significance of the manuscript is muted. In your methods section you indicate: ‘At the baseline and six-month visits, participants were asked about past six-month experience with police harassment motivated by racism or homophobia.’ You are measuring both racism and discrimination. However, in the text you only refer to ‘discrimination’. Please add racism e.g., racism and discrimination in those instances where discrimination is used. APA delineates between discrimination and racism: Racism is a form of prejudice that assumes that the members of racial categories have distinctive characteristics and that these differences result in some racial groups being inferior to others. Racism generally includes negative emotional reactions to members of the group, acceptance of negative stereotypes, and racial discrimination against individuals; in some cases it leads to violence. Discrimination refers to the differential treatment of the members of different ethnic, religious, national, or other groups. Discrimination is usually the behavioral manifestation of prejudice and therefore involves negative, hostile, and injurious treatment of members of rejected groups. <https://www.apa.org/topics/racism-bias-discrimination>

**Author response: We thank the reviewer for this important point and reference; discrimination encompasses a broad categorization where racial discrimination would be an aspect in addition to discrimination due to sexual orientation/sexual identity. We have updated and clarified the language in the manuscript to note that discrimination is referencing both racial and sexual identity discrimination. These updates appear in the introduction and discussion sections where we refer to these concepts.**

2. Also, I draw your attention to the following excerpt from an article by Lisa Bowleg on how to write about racism. She writes: ‘The language that researchers use shapes virtually every aspect of the research process. Consequently, these are not simply pedantic concerns. If researchers conceptualize “race” rather than social processes based on race (e.g., racism, racial trauma) as the source of health inequities, then every decision (e.g., the operationalization of variables, measures, hypotheses, analyses, interpretation of results, and the potential for intervention) hinges on this choice. It also detracts attention from more fundamental and modifiable factors such as those shaped by structural racism (e.g., occupation, household composition) that often provide greater explanatory power for racialized health inequities such as COVID-19 (coronavirus disease 2019) than “race” (see, e.g., Selden & Berdahl, 2020). Avoid euphemisms and other linguistic tropes that color-blind or otherwise erase the structural roots of racialized health inequities.’ (Please see for full article: https://journals.sagepub.com/doi/full/10.1177/10901981211007402). With that in mind, I wonder if the term ‘identity-based police harassment’ erases the state-sanctioned racialized violence aspects of these encounters and neutralizes its structural roots thus detracting from the fundamental thrust of your findings. Please (re)consider utilizing another term that is more precise in naming what this variable is measuring. In the survey it was referred to as ‘police harassment motivated by racism or homophobia’. Perhaps racialized police harassment and homophobia or some other appropriate descriptor?

**Author response: We thank the reviewer for this important distinction in language and have modified the wording in the manuscript to “racialized and homophobia-based police harassment (RHBPH)” instead of identify based police harassment (IBPH) which is much clearer in describing the police harassment identity factors that are part of the exposure.**

3. In the introduction on p. 3, the authors wrote: ‘Dual minority status—being both racial/ethnic minority as well as sexual minority—is thought to play a role in psychosocial vulnerability and adverse mental health outcomes, while network and structural factors also are implicated (7). Given the substantial health challenges experienced by Black sexual minority groups, there is a critical need to ensure adequate access to healthcare to ensure screening, and treatment and prevention to address mental health, substance use and related infectious disease risk.’ This second sentence is where you want to really hone in on what it is that you are hypothesizing. However, something seems to be missing. Suggested change: Given the enormous health disparities faced by Black sexual minority groups, there is a critical need to understand the impact of racism and discrimination on health outcomes to ensure adequate access to healthcare including screening, prevention, and treatment to address mental health, substance use and related infectious disease risk.’ But of course feel free to modify the language as you see fit.

**Author Response: We have modified this statement in the updated draft per the reviewer’s suggestion.**

4. In the methods section where you define Identity Based Police Harassment Exposure measure, please include an example of the question(s) from the survey.

**Author Response: In the survey, participants were asked if they were harassed by police; if they responded yes to this question, they were asked two follow up questions including if the harassment was due to their race, or due to their sexuality. The participants were allowed to answer yes to one or both of these questions. We have added this language to the methods section where we highlight the exposure variable.**

5. In the methods section, the authors wrote: We coded their responses into a three level ordinal exposure indicating increasing frequency of police harassment due to either racism or homophobia: level 0 indicates no such harassment at either time period (i.e., baseline or the six month follow-up visit), level 1 indicates that the participant reported IBPH at the baseline visit or at the six month follow-up visit, and level 2 indicates that the participant reported IBPH at both the baseline and six month follow-up visit. I’m curious about why you measured IBPH. Were respondents asked to indicate exposure by a yes/no response or where they asked to provide a frequency. Because the way it’s currently measured it may not fully capture the frequency or severity of the exposure. Let’s imagine a hypothetical case that respondents were asked to indicate how many exposures they had in the last 6 months. Respondent 1 responded 6 at baseline and 1 at 12 months, whereas Respondent 2 indicated 12 and zero. Based on the way you measured it, Respondent 1 would be placed in category 3 (7 exposures) and Respondent 2 in category 2 (12 exposures), although Respondent 2 had a greater number of IPBHs. Perhaps I’m misinterpreting this, but it seems at the very least the measure is imprecise and should be addressed in the limitations section.

**Author Response: The participants were not asked a frequency measure of the number of times the harassment occurred during the periods we measured; they were only asked if they had experienced any police harassment in the six months preceding the interview. The reviewer is correct that it is certainly possible that some participants experienced harassment more times than others; unfortunately, the questionnaire was not designed to capture this level of detail with respect to harassment due to race or sexual identity. In the limitations section we have added a note that our exposure does not capture the frequency of police harassment events.**

6. In Table 1, you use different language to label IBPH at 6mo vs 12mo. For example, at 6mo you use ‘no police harassment’ while at 12 mo ‘no IBPH’. Please select one or the other for consistency.

**Author Response: We thank the reviewer for catching this inconsistency and have modified the table to use consistent language using the updated exposure title of “racialized and homophobia-based police harassment”**

7. On p. 19 you note you controlled for a number of risky behaviors. I would recommend using risk factors instead of ‘risky behaviors’ as it has the potential to further stigmatize an already vulnerable population. Also, I’m not sure if I would include ‘demographics’ as a risky behavior. Please check.

**Author response: We have modified this language as well as removing the reference to any “risky behaviors” in the updated draft and refer to “factor” and “variable” instead where we present information on the statistical analysis.**

Reviewer #3: This paper addresses an important and novel research question about the relationship between police harassment and healthcare access among a multiply marginalized subpopulation of adults in the U.S. The introduction is concise yet builds a solid foundation for the study's results, the methods are sufficiently described (given the additional detail about HPTN 061 published elsewhere), the analysis and results are digestible, and the discussion couches findings within the context of existing literature with notes about analytic limitations and study implications. I do not have any suggested revisions for this manuscript.

**Author Response: Thank you for these comments We appreciate these positive comments on our manuscript.**

Reviewer #4: This is an interesting paper that focuses on an understudied issue - I think there is the potential for this manuscript to fill the gap in the literature but have several suggestions I'd like the authors to address to strengthen the paper.

OVERALL
- The rampant epidemic of stigma facing minoritized populations severely impacts all components of life. While this is especially highlighted at the beginning of the Intro, I think it would be an easy framing to really tie together both IBPH and medical mistrust/healthcare access. The two points are clearly tied, but I still think the argument presented is a bit weak.
- It's a disservice to talk about stigma and multiply minoritized individuals without talking about intersectionality.

**Author Response: Thank you for this suggestion. We have added language in the introduction to better frame this connection including “Additionally, it has been shown that for those that experience broader experiences of racism, sexual identity discrimination and harassment outside of the healthcare setting, these experiences can directly lead to mistrust in the healthcare settings (33). These negative social experiences which include RHBPH can not only shape health status, but also may influence healthcare encounters, including mistrust among doctors and clinicians in healthcare settings (34).” And “It should be noted, for instance, that excessive police violence may lead to individuals visiting the emergency department, where information would be collected on the circumstances of the injuries and may lead to further stigmatization of the individuals who experience these police related injuries (38, 39).”**

INTRODUCTION
- Page 4: need to add the word "of" - "...a study conducted in a sample 500 Black..."

**Author response: We thank the reviewer for the careful reading of the manuscript; we have made this correction in the updated draft**.

- Page 4, second paragraph: I'd be explicit about what is meant by IBPH. The reader can infer that this references any minoritized identity, but I think it's worth stating here since it's crucial to the entire paper.

**Author response: We have made clear what IBPH refers to by updating the phrasing to now read as “racialized and homophobia-based police harassment (RHBPH)” which we think it much more clear for the reader, and what has been suggested by another reviewer as a better way to identify the exposure for the paper.**

- Page 5, third paragraph: What about the fact that many BSMM don't even have a primary care physician? I'd think this is a bigger reason to go to the ED than intentionally avoiding their PCP.

**Author response: This is an excellent point; we have added this information to the introduction where we have expanded the language around the use of the ED.**

- Page 6, first paragraph: What about the role of health insurance? A lot of these factors may be directly due to lack of healthcare insurance, especially since this study took place prior to ACA.

**Author response: Our sample had approximately 60% of participants reporting health insurance coverage at baseline. It is possible that the due to 40% lacking health coverage, they may rely on certain medical services. We have therefore updated the introduction to include this point.**

METHODS
- The exact wording for the IBPH question should be provided. I assume separate questions were used for race and sexual orientation? If so, why were they collapsed for this paper? Did the question specifically say "homophobia?" If so, it might not resonate with participants who did not identify as gay.

**Author response: The exposure variable was defined in the paper to replicate the exposure that was used in other papers that were part of the HPTN061 body of literature. See “Feelemyer J, Duncan DT, Dyer TV, Geller A, Scheidell JD, Young KE, Cleland CM, Turpin RE, Brewer RA, Hucks-Ortiz C, Mazumdar M, Mayer KH, Khan MR. Longitudinal Associations between Police Harassment and Experiences of Violence among Black Men Who Have Sex with Men in Six US Cities: the HPTN 061 Study. J Urban Health. 2021 Apr;98(2):172-182. doi: 10.1007/s11524-021-00526-1. Epub 2021 Apr 5. PMID: 33821426; PMCID: PMC8079523” for one example where the exposure was similarly defined.**

- Why wasn't sexual orientation included as a potential covariate? This seems crucial for understanding sexual identity based police harassment - likely those who identified as bisexual or straight had different experiences than those who exclusively identified as gay.

**Author Response: We thank the reviewer for bringing up this important point. We did have one question that asked if participants had sex with men only, or if they had sex with men and women (the question asked was “do you have sex with men exclusively or do you have sex with men and women?). We included this as a covariate in the models. We have also added this variable to Table 1 where we list the demographic and other factors that were built into the multivariable models.**

- Page 8, Covariates: Why were some of these items not actually included in the analysis? A substantial chunk of HPTN 061 participants identified as something other than cisgender men, and trans individuals are known to be harassed by police and to avoid medical care due to transphobia. Neglecting to talk specifically about this subset is a major problem.

**Author Response: We controlled for transgender status in the multivariable models; we would note that transgender individuals were a small (less than 5%) part of the HPTN061 sample. We have added this information/data to Table 1. We would have liked to have done a separate analysis among transgender persons in the sample, but unfortunately did not have sufficient numbers to do this.**

- What was actually included in the multivariable models? Was it the list of "covariates" or was it the variables in Table 1?

**Author Response: All variables in Table 1 are included in the models; we have added this important point into the statistical analysis section. We have also updated Table 1 to include the breakdown of each variable/covariate with descriptive statistics for the entire sample at baseline and at the 12-month follow-up visit that was included in the multivariable model (including the variables for men who have sex with me vs men who have sex with men and women; and transgender individuals.**

RESULTS
- Why is depression mentioned here, but not in the Methods section? At the very least, the Methods should say that CES-D 10 was used.

**Author Response: We thank the reviewer for noting this omission in the methods. The CES-D scale was used for the depression variable, and is now listed in the methods section with a reference now included for the scale.**

- Definitions need to be provided for some of the less clear variables (either here or the Methods section) - specifically, health coverage, insufficient income, and unstable housing. Additionally, "STI (Any)" should indicate if it's self-report or tested, since HPTN 061 did STI testing.

**Author Response: We have updated the definitions for these specific variables in the methods section including lab testing for STI infection and other factors using the exact wording from the HPTN survey questionnaire. This update now appears in the methods in the section of “covariates” and is also spelled out in the Table 1 information.**

- Why was one of the IBPH levels "baseline or six months?" Only experiencing IBPH at baseline or at 6 months seems worthy of distinction, especially for longitudinal analyses. I recommend separating this into two categories unless the authors have good justification.

**Author response: As noted above, the exposure was measured this way to be consistent with other papers that have been published on the HPTN cohort that used this measure at these two time points in evaluating outcomes at the final (12 month) visit.**

- Table 2: what do the asterisks mean?

**Author Response: The asterisks have been removed from Table 2.**
